# Long-Read Assembly and Annotation of the Parasitoid Wasp *Muscidifurax raptorellus*, a Biological Control Agent for Filth Flies

**DOI:** 10.3389/fgene.2021.748135

**Published:** 2021-11-12

**Authors:** Xiao Xiong, Yogeshwar D Kelkar, Chris J Geden, Chao Zhang, Yidong Wang, Evelien Jongepier, Ellen O. Martinson, Eveline C Verhulst, Jürgen Gadau, John H Werren, Xu Wang

**Affiliations:** ^1^ Department of Pathobiology, College of Veterinary Medicine, Auburn University, Auburn, AL, United States; ^2^ School of Life Sciences and Technology, Tongji University, Shanghai, China; ^3^ Department of Biology, University of Rochester, Rochester, NY, United States; ^4^ Center for Medical, Agricultural and Veterinary Entomology, USDA Agricultural Research Service, Gainesville, FL, United States; ^5^ Department of Plastic and Reconstructive Surgery, Shanghai Ninth People’s Hospital, Shanghai Institute of Precision Medicine, Shanghai JiaoTong University School of Medicine, Shanghai, China; ^6^ Laboratory of Entomology, Wageningen University, Wageningen, Netherlands; ^7^ Institute for Biodiversity and Ecosystem Dynamics, University of Amsterdam, Amsterdam, Netherlands; ^8^ Department of Biology, University of New Mexico, Albuquerque, NM, United States; ^9^ Institute for Evolution & Biodiversity, University of Münster, Münster, Germany; ^10^ Alabama Agricultural Experiment Station, Center for Advanced Science, Innovation and Commerce, Auburn, AL, United States; ^11^ HudsonAlpha Institute for Biotechnology, Huntsville, AL, United States

**Keywords:** *Muscidifurax*, parasitoid wasp, biological control, housefly, linked-read technology, PacBio sequencing

## Abstract

The parasitoid wasp *Muscidifurax raptorellus* (Hymenoptera: Pteromalidae) is a gregarious species that has received extensive attention for its potential in biological pest control against house fly, stable fly, and other filth flies. It has a high reproductive capacity and can be reared easily. However, genome assembly is not available for *M. raptorellus* or any other species in this genus. Previously, we assembled a complete circular mitochondrial genome with a length of 24,717 bp. Here, we assembled and annotated a high-quality nuclear genome of *M. raptorellus*, using a combination of long-read (104× genome coverage) and short-read (326× genome coverage) sequencing technologies. The assembled genome size is 314 Mbp in 226 contigs, with a 97.9% BUSCO completeness score and a contig N50 of 4.67 Mb, suggesting excellent continuity of this assembly. Our assembly builds the foundation for comparative and evolutionary genomic analysis in the genus of *Muscidifurax* and possible future biocontrol applications.

## Introduction

*Muscidifurax* (Hymenoptera: Pteromalidae) is a chalcid wasp genus with nine characterized species, all of which are pupal parasitoids. *Muscidifurax raptor* was the first species described in the genus, in 1910 by Girault and Sanders (Girault and Sanders, 1910). In 1970, four sibling species were described: *M. zaraptor* Kogan and Legner, collected from the southwestern United States; *M. raptoroides* Kogan and Legner collected from Central America and Mexico; *M. raptorellus* Kogan and Legner collected from Uruguay and Chile; and a thelytokous species *M. uniraptor* Kogan and Legner collected from the central mountain range of the island of Puerto Rico (Kogan and Legner, 1970). Based on the mitochondrial gene sequence alignment in this genus, the most closely related sexual species to the asexual *M. uniraptor* is *M. raptorellus* (Taylor et al., 1997). Four additional *Muscidifurax* species were identified in China (Xiao et al., 2018).

*Muscidifurax raptorellus* (Chilean strain) is a gregarious parasitoid that typically produces 2–10 offspring per parasitized host pupa (Geden and Moon, 2009). The number of eclosed offspring depends on the host size (Seidl and King, 1993). The population found in Uruguay is partially gregarious (Legner, 1969). Females can lay 16–20 eggs per day during their peak ovipositional period and about 150 eggs during their lifetime (Petersen and Currey, 1996). In sharp contrast, *M. zaraptor* only deposits one egg per host, and the first larva will eliminate subsequent larvae or eggs deposited by superparasitism (McKay and Broce, 2004). *M. uniraptor* only produces a single female offspring from each host, and the parthenogenesis is caused by the infection of A strain *Wolbachia* bacteria (Zchori-Fein et al., 2000; Newton et al., 2016). The diverse reproductive strategies make this genus an excellent model system for the study of sexual vs. asexual evolution.

*M. raptorellus* is an effective biological control agent of dipteran filth flies, including house fly (*Musca domestica* L.), stable fly (*Stomoxys calcitrans* L.), horn fly (*Haematobia irritans* L.), black dump fly [*Hydrotaea aenescens* (Weidemann)], and flesh fly (*Sarcophaga bullata* Parker) (Petersen and Currey, 1996; Geden and Hogsette, 2006; Geden and Moon, 2009). Application of insecticide, which is the primary control strategy, is of limited effectiveness due to the evolution of resistant genes in these pests. Parasitoid wasps have great potential as an alternative management strategy that is more environmentally friendly and sustainable (Heraty, 2009). Wasps in the genus *Muscidifurax* are also of interest for comparative genomic studies, due to their close relationship to the model parasitoid genus *Nasonia*, which currently has genome assemblies for three species (Werren et al., 2010; Wang et al., 2020), with *Muscidifurax* estimated to be 15 million years divergent (Martinson et al., 2017a). Here, we report the first draft genome assembly of *M. raptorellus* using PacBio long-read sequencing. This well-assembled and annotated genome will provide an essential genetic toolkit for functional and evolutionary genomic studies in *M. raptorellus* and its sibling species. The high-quality reference genome could also inform and facilitate future genome manipulation in parasitoid wasps for more effective biological control strategies (Leung et al., 2020).

## Materials and Methods

### Sample Source and Insect Rearing

The source of *M. raptorellus* used in this study was derived from a colony maintained by Dr. Chris Geden at the Center for Medical, Agricultural and Veterinary Entomology, USDA Agricultural Research Service (Gainesville, FL). Genomic sequencing samples were collected from two independent colonies, both derived from the same USDA colony: one maintained at the Auburn University College of Veterinary Medicine in Auburn, Alabama, since 2019 (Aub sample) and the other one maintained at Koppert Biological Systems in the Netherlands (Kop sample) since 20 years ago. *M. raptorellus* was originally collected in 1965 from Chile but was referred to as *M. raptor* (Legner et al., 1967); subsequently described as *M. raptorellus* in 1970 (Kogan and Legner, 1970); and afterward distributed in North America for biological control efforts. The current colony was originally established from field-collected specimens on a New York poultry farm (Kaufman et al., 2001) and maintained in the Geden laboratory on housefly pupae. Samples from the colony were obtained from the Werren laboratory in 2016 and maintained on *Sarcophaga bullata* pupae and then sent to the Wang laboratory in Auburn, Alabama, in 2019 and maintained on commercial *Sarcophaga bullata* pupae (flesh fly pupae) at a constant temperature of 25°C and 24 h constant light. The Kop sample was maintained on *Lucilia* spp. pupae for 20 years and was sent to the Verhulst laboratory in 2014 and maintained on *Calliphora* spp. pupae at 25°C and 18 h/6 h light/dark conditions. Both the Aub and Kop samples were from the same fully inbred strain of *M. raptorellus*.

### Genomic DNA Extraction, Library Preparation, and Sequencing

High-molecular-weight (HMW) genomic DNA (gDNA) was extracted from adults of the *M. raptorellus* Aub sample using the Genomic-tip 20/G kit (Qiagen, Catalog No. 10223) with DNA concentration checked on a Qubit 3.0 Fluorometer (Thermo Fisher Scientific, United States). The size distribution and gDNA quality were assessed on an Agilent TapeStation 4200 machine (Agilent Technologies, CA) using the genomics kit (Agilent, Catalog No. 5067-5366). A total of 10 μg high-quality *M. raptorellus* genomic DNA was sheared into 20 kb fragments, and the end damage was repaired. After sequencing adapter ligation, the DNA fragment was annealed with Sequencing Primer v2 and Sequel II DNA Polymerase and bound to the SMRTbell templates, and the library was constructed following SMRTbell Template Prep Kit v2 following the CCS HiFi library protocol (Pacific Biosciences, CA). The size distribution of the constructed library was assessed using LabChip GX Touch HT (PerkinElmer, MA, United States), and the final library quantity was examined with a Qubit 3.0 Fluorometer (Thermo Fisher Scientific, United States). The PacBio library was sequenced on a PacBio Sequel II System at the HudsonAlpha Genome Sequencing Center (Supplementary Table S1).

HMW genomic DNA was diluted to ∼ 0.8 ng/μl with elution buffer for 10x Genomics library preparation using Chromium Genome Reagent Kit v2 (10× Genomics, Inc., CA). The diluted denatured gDNA, sample master mix, and gel beads were loaded to the genomic chip following the protocol and then ran on a 10× Chromium Controller to generate Gel Bead-In-EMulsions (GEMs). The obtained GEMs were used for the subsequent incubation and cleanup. The Chromium i7 Sample Index served as the library barcode to provide linked information. After quality control with a Qubit 3.0 Fluorometer (Thermo Fisher Scientific, MA, United States) and Agilent TapeStation 4200 (Agilent Technologies, CA), the 10× genomic sequencing was performed on an Illumina NovaSeq 6000 machine.

HMW gDNA was extracted from a pool of thirty females of the *M. raptorellus* Kop sample that were collected at the black pupal stage (∼16 days after egg-laying), using the Genomic-tip 100/G kit (Qiagen, Catalog No. 10243) combined with the Genomic DNA Buffer Set (Qiagen, Catalog No. 19060). The sample was ground to fine powder in liquid nitrogen by a plastic pestle, and the total DNA was extracted following the protocol provided by the manufacturer. After extraction, genomic DNA was sheared into 8–30 kb range by using g-TUBE (Covaris) following the manufacturer’s protocol. The quality and quantity of sheared genomic DNA were checked by gel electrophoresis with 1.5% TAE agarose gel stained with Midori Green (NIPPON Genetics) and by spectrophotometry (Nanodrop™ 2000, Thermo Fisher). The genomic DNA was measured and quality controlled at Novogene Co., Ltd. (Beijing, China). SMRTbell library templates were prepared for long-read sequencing on the PacBio Sequel system using three flow cells, to generate up to 70 kb long reads with an average read length of 12–15 kb. A total of 1.57 million high-quality subreads were obtained, with an estimated read depth of 55.8× (Supplementary Table S1).

### Genome Assembly, Polishing, and Assessment

The raw sequencing reads (Aub sample) from both PacBio library and 10× Genomics library were checked for sequencing quality using FastQC (Andrews et al., 2010) before genome assembly. *De novo* genome assembly for the *M. raptorellus* Aub sample was performed by a Supernova 2.1.1 (Weisenfeld et al., 2017) assembler using 400 million reads subsampled from the total amount of reads generated from the 10× Genomics library. Filtered HiFi PacBio reads were assembled by hifiasm v0.13 (Cheng et al., 2021) and HiCanu v2.1.1 (Nurk et al., 2020), dedicated assemblers using long-read sequencing. The Kop CLS PacBio data were assembled using Canu v2.1 (Koren et al., 2017). The Kop CANU assembly was polished with Pilon (version 1.22; parameter settings: fix = all) (Walker et al., 2014) to correct small errors based on high-quality 150 bp paired-end Illumina short reads (Table 1). A final round of polishing with Arrow (VariantCaller version 2.1.0) was performed to correct large structural errors, based on the raw PacBio reads that were aligned with Minimap2 (Li, 2018). Aub and Kop cultures have identical mitochondrial genomes (100% sequence identity) with only one 11 bp indel. The Aub 10× Genomics reads were aligned to the repeat-masked Kop assembly using the Longranger v2.1.6 (Zheng et al., 2016) software suite with the ALIGN pipeline. 58,350 SNPs were called by UnifiedGenotyper in the Genome Analysis Toolkit (GATK) (McKenna et al., 2010; DePristo et al., 2011). SNP positions in repetitive regions and variants outside the coverage depth threshold (120–500 bp) were filtered out using BEDTools v2.30.0 (Quinlan, 2014). A total of 11,523 homozygote SNPs between Aub and Kop were identified, and the percentage of fixed differences in the nuclear genome was estimated to be 0.0038%. To achieve the best assembly, these draft assemblies with different assemblers from both Aub and Kop samples were merged into a draft assembly using an assembly combination tool quickmerge v0.3.0 (Chakraborty et al., 2016). Potential bacterial contaminations were checked using a pipeline described in our previous research (Wang et al., 2020), and no bacteria contig contamination was discovered. The draft assembly was polished to yield a final high-quality assembly with the 10× Genomics Illumina short reads for indel correction using Pilon v1.23.0 (Walker et al., 2014). The final genome assembly was evaluated based on the N50 size of contigs and RNA-seq read mapping percentages, and genome completeness was assessed by BUSCO version 4.0.6 (Seppey et al., 2019). The BUSCO scores were calculated using arthropoda_odb10 with a total of 1,013 orthologs.

**TABLE 1:** Summary statistics of the *Muscidifurax raptorellus* genome assemblies.

Genome assembly	Aub_hifiasm	Aub_HiCanu	Kop_PacBio	Final
Data and coverage				
PacBio sequencing data	15.0 Gb Sequel II CCS reads	15.0 Gb Sequel II CCS reads	17.7 Gb Sequel CLRs	-
Illumina sequencing data	81.6 Gb	81.6 Gb	20.6 Gb	-
Genome coverage	CCS: 48×, Illumina: 260×	CCS: 48×, Illumina: 260×	CLS: 56×, Illumina: 66×	PacBio: 104×, Illumina: 326×
**Assembly statistics**				
Genome size (bp)	315,727,724	316,569,142	316,926,883	313,931,273
No. of scaffolds	489	527	384	226 + chrM
Scaffold N50 (bp)	1,479,014	2,597,351	2,784,708	4,673,378
Contig N50 (bp)	1,479,014	2,597,351	2,784,708	4,673,378
Maximum contig length (bp)	8,668,935	14,498,644	14,510,203	21,163,931
**Completeness**				
BUSCO completeness	97.90%	97.90%	97.90%	97.90%
Single-copy BUSCO	95.90%	95.90%	96.20%	96.80%
Duplicated BUSCO	2.00%	2.00%	1.70%	1.10%
Fragmented BUSCO	0.50%	0.50%	0.50%	0.50%
Missing BUSCO	1.60%	1.60%	1.60%	1.60%
**Mapping statistics**				
% of gDNA-seq reads mapped	96.67%	96.74%	96.71%	96.68%
% of gDNA-seq covered positions	99.99%	99.91%	99.78%	99.94%
Adult RNA-seq, all mapped	97.41%	97.49%	97.42%	97.24%
Adult RNA-seq, uniquely mapped	95.02%	94.78%	95.19%	94.56%

### RNA-Seq Data Processing and Transcriptome Assembly

Total RNA was isolated from adult whole-body samples of adult male and female *M. raptorellus* in three biological replicates for each sex from samples collected in the Werren laboratory. The RNA extraction, quantification, library preparation, and sequencing protocol were performed as previously described (Martinson et al., 2017b). A total of 308,475,537 reads were obtained from six samples. FastQC (Andrews et al., 2010) was used for quality control of raw RNA-seq data. The paired-end RNA-seq reads were processed with Trimmomatic v0.38 (Bolger et al., 2014). After trimming the potential adapter sequences, we performed *de novo* assembly of the *M. raptorellus* transcriptome using Trinity v2.4.0 (Haas et al., 2013), and pre-aligned transcripts were annotated by Cufflinks v2.2.1 (Trapnell et al., 2012).

### Repeat Annotation

A *de novo M. raptorellus* repeat database was constructed using RepeatModeler v2.0.1 (Flynn et al., 2020) with the default parameters, which employs three complementary computational programs, RECON v1.0.8 (Bao and Eddy, 2002), RepeatScout v1.0.5 (Price et al., 2005), and Tandem Repeats Finder (TRF) (Benson, 1999), to annotate repetitive elements in our genome assembly. RepeatScout is a *de novo* repeat finder to identify highly conserved repetitive elements, while RECON can find less conserved elements. TRF is a program to locate and display tandem repeats. The high-quality library of transposable element (TE) families was then used to mask homologous repeats and low-complexity DNA sequences using RepeatMasker v4.0.6 (Chen, 2004) with RMBlast v2.10.0 as the default search engine.

### Gene Prediction and Functional Annotation

To annotate the structures and functions of the *M. raptorellus* genome, we integrated *ab initio* and RNA-seq based methods to predict the genes in repeat-masked assembly. For RNA-seq prediction, the trimmed RNA-seq reads were aligned to the repeat-masked genome assembly using Tophat v2.1.1 (Kim et al., 2013) and then assembled into transcripts using cufflinks v2.2.1 (Trapnell et al., 2012) with default parameters. In addition, *de novo* assembly of *M. raptorellus* transcriptomes was achieved by Trinity v2.4.0 (Haas et al., 2013). The annotation of the genome assembly was performed using the MAKER v2.31.9 (Cantarel et al., 2008) annotation pipeline. Gene models were predicted using *ab initio* gene prediction algorithms with protein and transcriptome evidence by EST2GENOME and PROTEIN2GENOME procedures in MAKER (Data S1). The generated GFF3 file and assembled transcriptome from RNA-seq prediction were provided as expressed sequence tag (EST) evidence. The Arthropoda_odb10 dataset served as protein homology evidence. After evaluation and filtering with evidence scores, the predicted genes were used to train both SNAP (Korf, 2004) and AUGUSTUS (Stanke and Waack, 2003; Stanke et al., 2006) gene predictors. Two additional iterations were performed to generate the final predicted gene models for the *M. raptorellus* genome. A homology-based gene prediction tool, Gene Model Mapper (GeMoMa) (Keilwagen et al., 2019), was also utilized to annotate the coding genes in *M. raptorellus* using well-annotated *Nasonia vitripennis* OGS2 (official gene set 2) (Rago et al., 2016) as the protein reference.

### Comparative Genome Analysis

To compare the genome structure between *M. raptorellus* and *N. vitripennis* genomes, the homologous regions in these two genomes were identified using MCScanX (Wang et al., 2012) with default parameters, which is a Python package for synteny detection and evolutionary analysis. The inferred gene pairs and linked relationships were visualized and placed in the context of whole-genome collinearity using a genomic circle generated by Circos (Krzywinski et al., 2009). The chromosome-level genome assembly of *N. vitripennis* (Nvit_psr_1.1) (Dalla Benetta et al., 2020) was downloaded at NCBI Assembly with accession number GCA_009193385.2.

### Phylogenetic Analysis

To investigate the phylogenetic relationship between *M. raptorellus* and other Hymenoptera insect species, nine representative species (jewel wasp *Nasonia vitripennis*, honey bee *Apis mellifera*, turnip sawfly *Athalia rosae*, fig wasp *Ceratosolen solmsi marchali*, Indian jumping ant *Harpegnathos saltator*, Braconid wasp *Microplitis demolitor*, wood wasp *Orussus abietinus*, red paper wasp *Polistes canadensis*, and minute polyphagous wasp *Trichogramma pretiosum*) were selected from 40 Hymenoptera species in OrthoDB v10.1 (https://www.orthodb.org/) (Kriventseva et al., 2018). A total of 4,390 1:1 single-copy orthologs among these nine genomes were identified. The protein sequences for *M. raptorellus* were aligned to *N. vitripennis* using BLASTp alignments with a minimum of 60% sequence identity, and 3,662 1:1 orthologs were identified. The detailed information of 3,662 1:1 single-copy genes in the *M. raptorellus* genome and the nine representative Hymenoptera genomes is provided in Data S1. Subsequently, the protein sequences of the single-copy orthologs in the nine species were extracted from the OrthoDB fasta file, and *M. raptorellus* protein sequences of these genes were extracted from our genome assembly. The protein sequences across the selected Hymenoptera species and *M. raptorellus* were independently aligned with MAFFT v7.407 (Katoh and Standley, 2014). The protein alignments were concatenated for phylogenomic analysis. ProtTest 3 (Darriba et al., 2011) was used to estimate the best protein model of protein evolution. The maximum-likelihood (ML) phylogenetic tree was finally built with the concatenated protein sequence by using RAxML v8.2 (Stamatakis, 2014) with the best JTT protein model. 1,000 rapid bootstrap replicates were applied for evaluation of their branch supports. The tree was displayed by FigTree v1.4.4 (http://tree.bio.ed.ac.uk/software/figtree/).

## Results and Discussion

### Genome Assembly and Assessment

Two independent PacBio libraries were constructed for the assembly of *M. raptorellus* genome (see *Materials and Methods*). The PacBio Sequel II HiFi reads (14,992,520,996 bp) generated from the Aub sample were assembled using hifiasm and HiCanu, and the Kop PacBio data (17,675,696,457 bp) were assembled using Canu (see *Materials and Methods*). The genome size of all three assemblies ranges from 315.7 to 316.9 Mbp (Table 1), which is very close to the estimated size from 10× Genomics data using Supernova based on K-mer profiles (315 Mbp), indicating high confidence in the genome size. The merged genome has significant improvement over individual assemblies, in terms of reduction in the number of contigs (from 527 to 226), the increase in contig N50 (from 1.5 to 4.7 Mb), and the maximum contig length (from 8.7 to 21.2 Mb), as well as a reduced proportion of duplicated BUSCO (from 2 to 1.1%; Table 1), without sacrificing the DNA and RNA sequencing mapping rate (Table 1). The final assembled genome is 313,931,273 bp in length with 226 scaffolds (the GC content is 40.06%) and a circularized mitochondrial genome (GenBank accession number MT985329) (Lin et al., 2021). The contig N50 is 4,673,378 bp, and the BUSCO completeness score is 97.9% (96.8% single-copy, 1.1% duplicated, 0.5% fragmented, and 1.6% missing). The adult RNA-seq reads were aligned to the *M. raptorellus* assembly using Tophat (Trapnell et al., 2009), and 97% of the reads were mapped to the genome. The 10× Genomics short-read data were also mapped to the genome assembly, and the alignment rate was 96.68%. The proportion of the genome with zero depth was 0.06%. The assembly and mapping statistics suggest that the quality of our assembly is high in both genome completeness and continuity (Table 1).

### Syntenic Analysis With *Nasonia vitripennis* Genome

**FIGURE 1:**
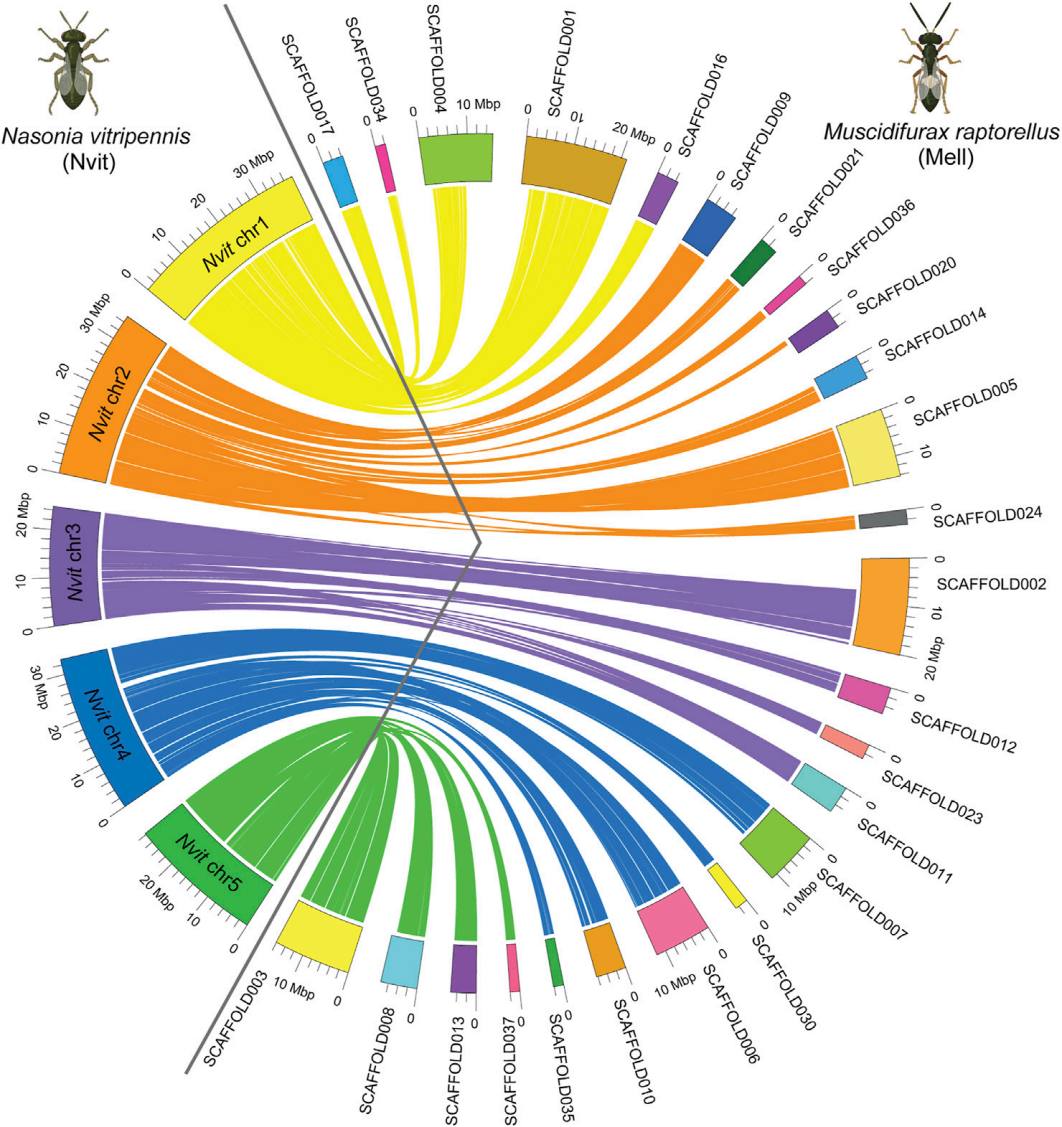
Genome comparisons between *Muscidifurax raptorellus* and *Nasonia vitripennis*. A total of 25 largest scaffolds in the *M. raptorellus* assembly showed a one-to-one relationship with the five chromosomes in the *N. vitripennis* genome. Chrs 1–5 on the left of the circle represent *N. vitripennis* chromosomes, and scaffolds on the right represent *M. raptorellus* assembled scaffolds. Parts of the figure were created with BioRender.com.

*N. vitripennis* and the congeners of *M. raptorellus*, *M. uniraptor*, and *M. zaraptor* have a haploid karyotype of n = 5 (Gokhman and Westendorff, 2000; Goodpasture, 1974; Silva-Junior et al., 2000). A total of 25 scaffolds from our *M. raptorellus* assembly with a total length of 187.4 Mb (59.7% of the whole assembly) were unambiguously aligned to the five assembled chromosomes in the *N. vitripennis* genome (Figure 1). The *N. vitripennis* chromosome assembly was based on recombination data between two closely related species (*N. vitripennis* and *N. giraulti*) (Niehuis et al., 2010; Desjardins et al., 2013), with all non-repetitive and non-centromeric regions correctly assembled and oriented (total chromosome size 159.4 Mb, 55% of the genome). The remaining 40% repetitive regions (Table 2) were not assembled into *N. vitripennis* chromosomes. The majority of *N. vitripennis* chromosomal regions have a collinearity relationship with *M. raptorellus* scaffolds (Table 1), suggesting high evolutionary conservation. The synteny analysis results also identified regional inversion, translocation, and duplication events, which will shed light on the genome evolution in these two genera.

**TABLE 2:** Summary repeat element classes in *Muscidifurax raptorellus* and *Nasonia vitripennis* genomes.

	*Muscidifurax raptorellus*		*Nasonia vitripennis*	
	# of elements	Length (%)	# of elements	Length (%)
**Retroelements**				
Penelope	913	327,347 (0.1%)	1,065	317,344 (0.11%)
LINEs	17,663	18,752,397 (5.97%)	14,783	14,534,403 (5.07%)
L2/CR1/Rex	7,925	7,800,224 (2.48%)	6,577	5,837,873 (2.03%)
R1/LOA/Jockey	6,013	6,346,905 (2.02%)	4,008	3,617,626 (1.26%)
R2/R4/NeSL	0	0 (0%)	151	406,741 (0.14%)
**LTR elements**				
BEL/Pao	1,496	1,683,607 (0.54%)	883	993,809 (0.35%)
Ty1/Copia	1,992	1,660,186 (0.53%)	2,396	2,624,950 (0.91%)
Gypsy/DIRS1	17,473	22,464,754 (7.16%)	9,516	9,681,184 (3.37%)
**DNA transposons**				
hobo-Activator	5,99	261,203 (0.08%)	646	248,902 (0.09%)
Tc1-IS630-Pogo	5,037	2,550,239 (0.81%)	3,453	4,340,897 (1.51%)
PiggyBac	443	257,410 (0.08%)	549	293,323 (0.1%)
Tourist/Harbinger	115	54,423 (0.02%)	61	35,468 (0.01%)
Rolling-circles	2,391	1,701,169 (0.54%)	5,841	2,970,560 (1.04%)
**Unclassified**	136,718	55,208,650 (17.59%)	136,074	63,582,769 (22.16%)
**Simple repeats**	150,695	6,103,642 (1.94%)	132,857	5,673,959 (1.98%)
**Low complexity**	10,350	497,537 (0.16%)	8,588	400,956 (0.14%)
**Total**	359,224	125,669,693 (40.03%)	327,448	115,560,764 (40.27%)

### Repeat Annotation

Repetitive regions accounted for 40% of the *M. raptorellus* genome with a total length of 126 Mbp based on the *M. raptorellus* specific repeat database (Table 2). The proportion of repeat regions is similar to that in *Nasonia vitripennis*, a jewel wasp species in the *Nasonia* genus (40.27%). LINEs (6.0%) and Gypsy (7.2%) elements are the most abundant classes in *M. raptorellus*, both with significantly higher abundance compared to those in *N. vitripennis* (Table 2).

### Phylogeny With Hymenopteran Genomes

**FIGURE 2:**
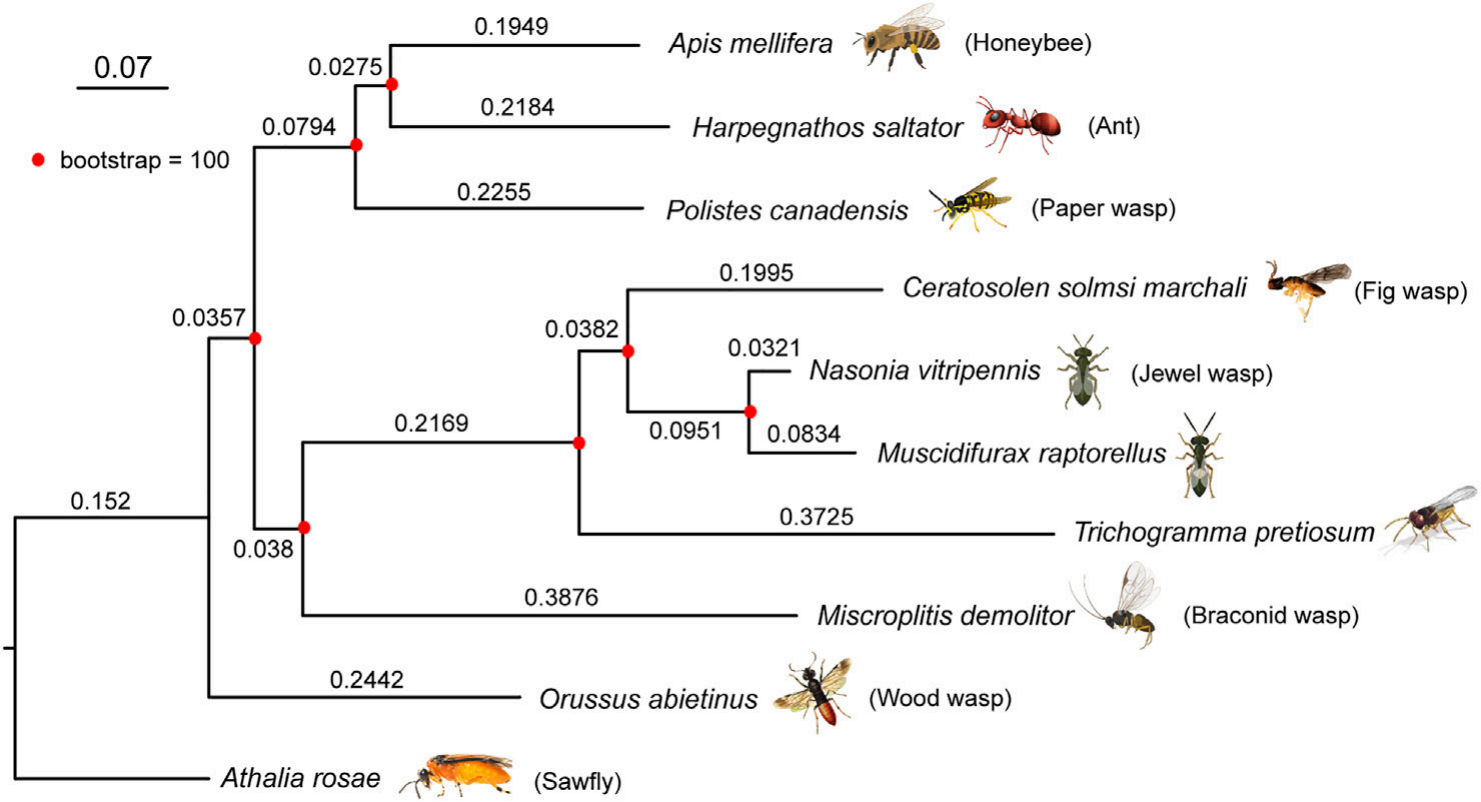
Phylogenetic relationship between *M. raptorellus* and nine representative hymenopteran species. A maximum-likelihood phylogenetic tree of *M. raptorellus* with nine other hymenopteran species was constructed based on 3,662 shared 1:1 single-copy proteins, using RAxML v8.2. The sawfly *Athalia rosae* was used as the outgroup. The bootstrap values were supported at 100/100. The length of each branch is shown on the branches. Parts of the figure were created with BioRender.com.

To construct the phylogenetic tree of *M. raptorellus* and other hymenopteran species, we used 3,662 single-copy 1:1 orthologs in nine species (turnip sawfly, parasitic wood wasp, Braconid wasp, minute polyphagous wasp, jewel wasp, fig wasp, paper wasp, ant, and honey bee). *M. raptorellus* clustered with the chalcid wasp species within the superfamily Chalcidoidea (Figure 2). *M. raptorellus* is the closest outgroup species to the jewel wasp *Nasonia* genus that has a high-quality reference genome, which will facilitate the evolutionary studies in the *Nasonia* subgroup and parasitoid wasp comparative genomics.

### Gene Annotations

After repeat regions were soft-masked, the first-round MAKER annotation based on Trinity output generated 18,392 gene models (Supplementary Data S2). Subsequent MAKER iterations resulted in 10,362 protein-coding genes supported by both RNA-seq and gene prediction algorithms (Supplementary Data S2). Among them, 7,520 single-copy orthologs were identified between *M. raptorellus* and *N. vitripennis* (Supplementary Data S3). To evaluate the completeness and quality of predicted genes, we compared the gene length distributions of the 7,520 orthologs and found an average CDS length of 1,008 bp in *M. raptorellus* (standard deviation = 1,585) and 1,035 bp in *N. vitripennis* (standard deviation = 1,631). The 3,662 single-copy 1:1 orthologs between *M. raptorellus* and nine other hymenopteran species also have similar CDS length distributions (Supplementary Figure S1), indicating good gene model quality for these orthologs in *M. raptorellus*. To perform the gene annotation using an independent approach, 9,520 protein-coding genes (with 20,493 transcript isoforms) were annotated using the homology-based gene predictor GeMoMa (Keilwagen et al., 2016; Keilwagen et al., 2018) (Supplementary Data S2). 417 tRNA (transfer RNA) genes and 83 rRNA (ribosomal RNA) gene clusters were also annotated in the genome (Supplementary Data S4).

## Data Availability

The sequencing data generated for this study can be found in the NCBI Sequence Read Archive database with accession number SRR15058746. The draft genome assembly of *M. raptorellus* has been deposited at NCBI under Assembly accession number JAHUUD000000000. Supplementary Material is available at github.com/XuWangLab/MellV1_genome_assembly.
