# Supplementary material for: Long-Read Assembly and Annotation of the Parasitoid Wasp Muscidifurax raptorellus, a Biological Control Agent for Filth Flies
Source: Front Genet. 2021 Nov 12;12:748135. doi: 10.3389/fgene.2021.748135 (PMC8633841; doi:10.3389/fgene.2021.748135)

Figure S1. Comparison of the gene length distributions for 3,662 shared 1:1 single copy orthologs in *M. raptorellus* and nine representative hymenopteran species.

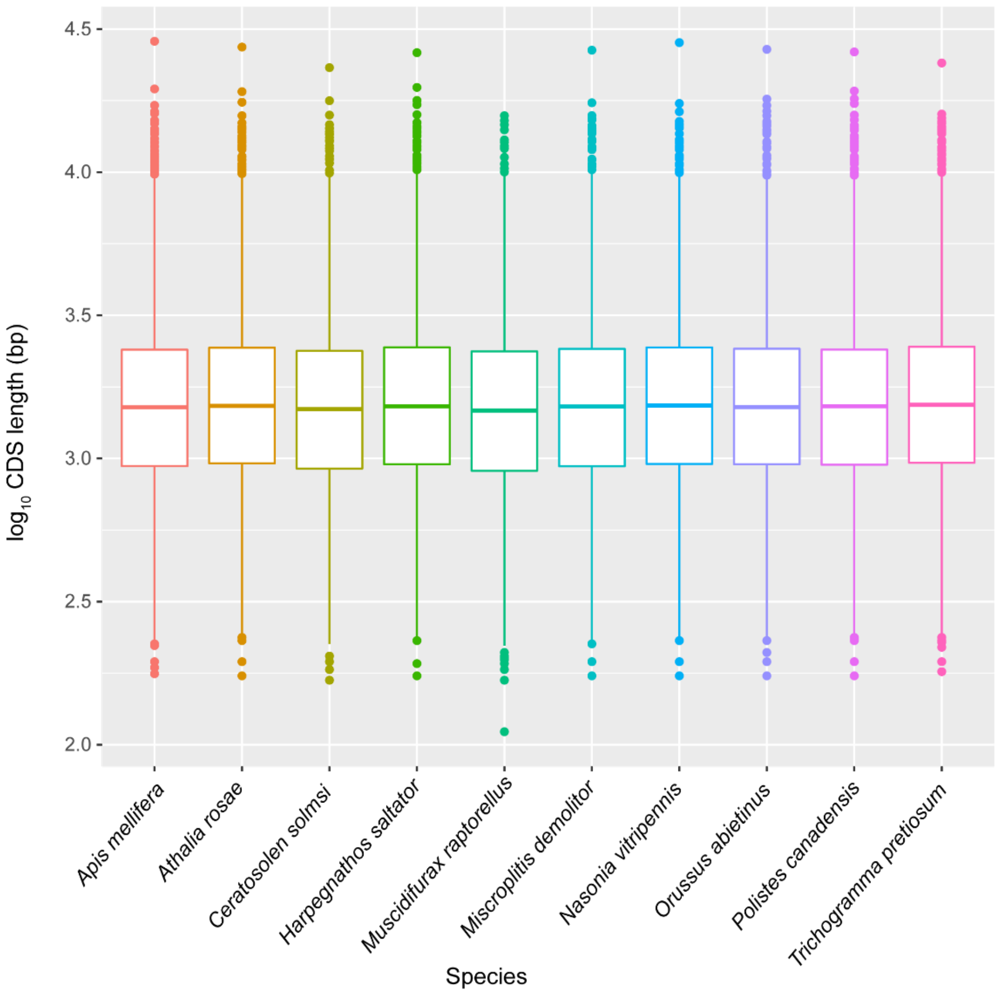

Supplement: Supplementary file 1 [file DataSheet2.PDF]
