# Supplementary material for: Long-Read Assembly and Annotation of the Parasitoid Wasp Muscidifurax raptorellus, a Biological Control Agent for Filth Flies
Source: Front Genet. 2021 Nov 12;12:748135. doi: 10.3389/fgene.2021.748135 (PMC8633841; doi:10.3389/fgene.2021.748135)
Supplement: Supplementary file 2 [file DataSheet1.PDF]

Table S1. Summary of Aub and Kop PacBio read statistics.

Statistics	Aub (CCS)	Kop (CLS)
Final library size	20 Kb	8-30 Kb
Total raw bases	268 Gb	18 Gb
Single pass bases	61 Gb	-
Processed CCS/CLR bases	14,992,520,996 bp	17,675,696,457 bp
Number of CCS/CLR reads	1,097,110	1,569,719
CCS/CLR longest read	32,312 bp	75,333 bp
CCS/CLR read N50	18,653 bp	18,284 bp
CCS/CLR read N90	13,608 bp	6,520 bp
